# Home-range use patterns and movements of the Siberian flying squirrel in urban forests: Effects of habitat composition and connectivity

**DOI:** 10.1186/s40462-016-0071-z

**Published:** 2016-02-17

**Authors:** Sanna Mäkeläinen, Henrik J de Knegt, Otso Ovaskainen, Ilpo K Hanski

**Affiliations:** Finnish Museum of Natural History LUOMUS, University of Helsinki, P. O. Box 17 (P. Rautatiekatu 13), Helsinki, FI-00014 Finland; Department of Biosciences, University of Helsinki, P. O. Box 65 (Viikinkaari 1), FI-00014 Helsinki, Finland; Current address: Resource Ecology Group, Wageningen University, Droevendaalsesteeg 3a, Wageningen, 6708 PB The Netherlands

**Keywords:** Connectivity, Habitat fragmentation, Home range, Movements, Nest-site use, Siberian flying squirrel, Urbanization

## Abstract

**Background:**

Urbanization causes modification, fragmentation and loss of native habitats. Such landscape changes threaten many arboreal and gliding mammals by limiting their movements through treeless parts of a landscape and by making the landscape surrounding suitable habitat patches more inhospitable. Here, we investigate the effects of landscape structure and habitat availability on the home-range use and movement patterns of the Siberian flying squirrel (*Pteromys volans*) at different spatial and temporal scales. We followed radio-tagged individuals in a partly urbanized study area in Eastern Finland, and analysed how landscape composition and connectivity affected the length and speed of movement bursts, distances moved during one night, and habitat and nest-site use.

**Results:**

The presence of urban habitat on movement paths increased both movement lengths and speed whereas nightly distances travelled by males decreased with increasing amount of urban habitat within the home range. The probability of switching from the present nest site to another nest site decreased with increasing distance among the nest sites, but whether the nest sites were connected or unconnected by forests did not have a clear effect on nest switching. Flying squirrels preferred to use mature forests for their movements at night.

**Conclusions:**

Our results suggest that the proximity to urban habitats modifies animal movements, possibly because animals try to avoid such habitats by moving faster through them. Urbanization at the scale of an entire home range can restrict their movements. Thus, maintaining a large enough amount of mature forests around inhabited landscape fragments will help protect forest specialists in urban landscapes. The effect of forested connections remains unclear, highlighting the difficulty of measuring and preserving connectivity in a species-specific way.

**Electronic supplementary material:**

The online version of this article (doi:10.1186/s40462-016-0071-z) contains supplementary material, which is available to authorized users.

## Background

Anthropogenic habitat changes can affect animal populations in several ways; for example by reducing habitat availability, or through impeding both daily and dispersal-related movements, thereby reducing interactions among individuals and, consequently, genetic exchange [1]. One of the key interests when studying animal movements is to find out how organisms respond to their environment, and changes therein [2]. Given that urbanization is considered to be a major threat for vertebrate species and that the rate of urban expansion is accelerating worldwide [3, 4], more research and conservation efforts should be targeted at species living in these human-modified environments [5]. The biggest threats of urbanization to wildlife are caused by the modification, fragmentation and loss of native habitats [6, 7]. Urban landscapes are often spatially complex mosaics, leaving remnants of native habitats surrounded by different kinds of new habitat types. They are also characterized as having a highly variable landscape between native patches, possibly with movement barriers such as wide roads or densely built residential areas [8]. While some species have shown behavioural plasticity and have adapted to inhabit urban areas [9, 10], moving in human-modified landscapes has been considered costly and risky for species that are adapted to live in formerly continuous landscapes [11].

Means to conserve species in modified landscapes have included management of the remaining native habitat and preserving movement corridors between habitat patches to maintain connectivity [12–14]. Measures of functional connectivity, that take into account species-specific movement abilities, have also been considered important [15, 16]. However, the presence of movement corridors and the configuration of the landscape have had varied effects on species [17, 18]. For example, results on corridor use of different taxa are conflicting, partly because the utility of corridors is species-specific and depends on the width and structure of the corridor [19–21]. Recent studies have also indicated that improvement of the quality of the habitat between suitable patches can in some cases be a more cost-effective conservation option than, for example, the construction of corridors or management of the remnant habitat patches [17, 22, 23].

The long-term existence of any species within continuously changing and expanding urban areas is related to its ability to exploit remaining habitat fragments, its responses to edges, and its willingness to cross gaps and use the landscape matrix between suitable patches [24, 25]. Here, matrix is defined as the interspersed landscape area between the patches of suitable habitat (such as mature forest fragments). Species have been found to be neutral, positive or negative regarding their use of matrix to move between the suitable habitat patches, for example, showing no resistance to use matrix, moving quickly through areas where the crossing distance is smaller than a particular threshold or being reluctant to enter the area between habitat patches [26–28]. Arboreal mammals, generally considered susceptible to changes in their native habitats, can serve as good model organisms to study movement behaviour in human-modified landscapes. Many of them are threatened by urban sprawl, habitat fragmentation and loss due to their strict habitat requirements, limited movement abilities and possible reluctance to move through the matrix [29, 30]. A special group are gliding species, whose movements through fragmented landscapes are constrained by maximum gliding distances [28].

The Siberian flying squirrel (*Pteromys volans*, hereafter flying squirrel) is an arboreal rodent inhabiting the Eurasian boreal forest zone, and its distribution extends from Finland and Estonia through the Asian continent all the way to Japan and the Korean peninsula [31]. Within the European Union the flying squirrel is classified as vulnerable, and the population in Finland has been declining due to destruction and fragmentation of suitable habitat caused by forest management [32, 33]. The most suitable breeding habitat for the flying squirrel is spruce-dominated mature boreal forest with a mix of deciduous trees that provides food and nesting cavities [34, 35]. In addition to mature spruce forests, flying squirrels use younger forests for foraging and moving [36]. Flying squirrels are highly dependent on trees and move almost exclusively by gliding from tree to tree. Gaps wider than few meters are crossed by climbing to the top part of the nearest tree and gliding over the gap. Through gliding they are able to cross relatively narrow (30–50 m) treeless gaps [37]. Females occupy home ranges of ca 8 ha usually located within one suitable forest patch. Males occupy large home ranges of ca 60 ha that often include several female home ranges and several forest patches, and consequently they need to move longer distances than females [38, 39]. Within their home ranges, flying squirrels have several nests between which they frequently change. These consist of tree cavities, twig nests built by the red squirrel (*Sciurus vulgaris*) and nest boxes, out of which the females prefer cavities during the breeding season [40]. Flying squirrels are nocturnal, thus movements consist of night-time activity periods interrupted by daytime resting in a nest. During one night, an individual typically makes several bursts of movements interrupted by staying in a nest or feeding [38].

Despite its preference for mature and relatively undisturbed forest, flying squirrels have also been found to inhabit forest patches near human settlements [41]. Consequently, the expansion of urban infrastructure and the strict legal protection of the species have created conflicts in many areas across Finland. In addition, recent studies have shown that legal protection of the species is inefficient due to the limited size of the protection areas, which cover only a small part of a home range [42, 43]. As earlier studies have been restricted to managed forests outside cities, and because little is known on the behaviour of the species in urbanized areas, there is an urgent need to increase our understanding of its habitat use in relation to urban landscape.

In this paper, we investigate the influence of urban landscape at different spatio-temporal scales on home-range use and movements of the Siberian flying squirrel. At the smallest scale, we ask how the distance travelled and speed during a single movement burst are related to small-scale habitat composition along that burst. At the scale of one night, we ask how the total distance moved depends on habitat composition within the home range, on the season, on the sex of the individual, and whether it varies among individuals within the sexes. We then investigate how the number of distinct nest sites is related to home-range size and habitat composition within the home range, and how individuals use habitats during movement bursts. Finally, we view home-range movements as movements among the network of nest sites, and examine how the choice of the next nest site, relative to the current position of the individual, depends on the distances between the nest sites and on the habitat composition and connectivity between the nest sites. In particular, we ask if the existence of forested connections influences the order in which the individuals visit the nest sites, and if the observed patterns differ between the sexes. Based on earlier results, we hypothesize that males respond to habitat composition so that they will move longer distances if there is less suitable habitat, whereas we expect females to move mostly within a single forest patch of suitable habitat. We also hypothesize that flying squirrels avoid moving through urban areas. We expect that the movement probability between consecutive nest sites increases with increasing physical and functional proximity, the latter measuring connectivity through forested habitats without gaps wider than 50 m.

## Methods

### Study area and habitat classification

**Fig. 1:**
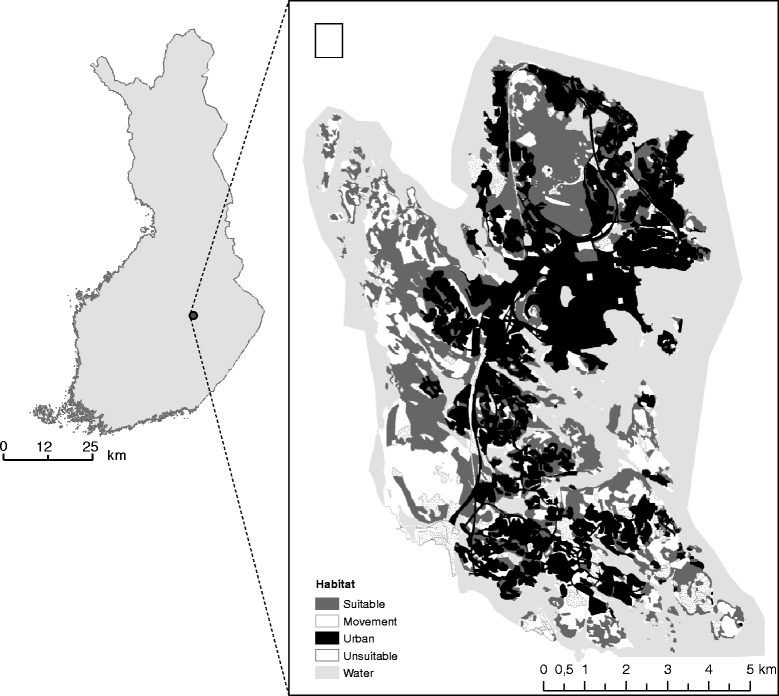
Map and habitat classification of the study area in Kuopio, Eastern Finland. Habitats are classified by their suitability for the flying squirrel. Suitable habitat (H1) denotes mature spruce-dominated forests. Movement habitat (H2) consists of forests that are over 10 m in height. Urban habitat (H3) consist of residential areas, roads or other habitats dominated by human land use. Clear cut areas, fields and sapling stands are combined to unsuitable habitat (H4). Water bodies (H5) are not utilized by the species and may form barriers for movements

The study was conducted in the city of Kuopio, Eastern Finland (62° 53’N, 27° 41’E) in 2008–2012. The study area of 73 km^2^ is dominated by forests (52 %) and urban habitats (38 %), but also includes clear cut areas and cultivated fields (5 %), sapling stands (3 %) and water bodies (3 %). The main tree species occurring in the study area are Norway spruce (*Picea abies*), Scots pine (*Pinus sylvestris*) and silver and downy birch (*Betula pendula, B. pubescens*). The study area belongs to Northern Savo core area of herb-rich vegetation and about one third of the forests are covered by groves or heaths with rich grass-herb vegetation [44]. With the help of aerial photographs, forest stand information received from the City of Kuopio and an earlier field survey [41], we classified the study area according to habitat suitability for the flying squirrel (Fig. 1), using ArcGIS 10 (Esri): H1) suitable habitat (spruce-dominated mature forests with deciduous trees such as aspen *Populus tremula*, and alders *Alnus incana* and *A. glutinosa*, i.e., the main breeding habitat of flying squirrel), H2) movement habitat (other forest types with height over 10 m, for example pure birch, pine or spruce forests), H3) urban habitat (old and recently developed residential areas, also residential areas with trees of varying species composition), H4) unsuitable habitats (clear cut areas, fields and sapling stands) and H5) water bodies.

### Collection of radio-tracking data

In total 19 adult females and 22 adult males were captured from nest boxes or trapped from nesting cavities and fitted with radio collars that weighed 5 g (Biotrack, U.K.). Radio tracking was conducted from the beginning of March until the end of September in 2008–2012. All individuals were located at least once a week in daytime to find their daily nest sites. Night-time movements of two females were not monitored, but their daytime locations were used in modelling movements between nest sites. In order to explore the movements and corridor use, we followed individuals for 3–5 nights per week in a continuous fashion, mostly for 30–150 min at a time. We tracked the animals by foot and recorded the tree or tree group every time the individual changed its location. The duration of the time the squirrel was not moving (e.g., while foraging or in a nest) was also measured. In early spring and late fall (March and September, respectively) the movements were followed mainly at the time of highest activity (early night), but during all the other months we also followed movements in the early morning. The relevant data (including spatial coordinates, times, used tree species and possible visual behavioural observations) of night-time tracking periods and those of daily nest sites were saved in a GPS device (Trimble Juno SB handheld).

We obtained data on movement tracks of 17 females and 22 males (for a more detailed description, see Additional file 1), of which five individuals were followed in several years. In total, females were followed for 378 h and males for 556 h. Tracking duration varied between 31–246 min, and average nightly tracking times were 94.5 (± SE 2.5) min and 107.2 (± SE 2.0) min for females and males, respectively. Moved distance per nightly tracking period varied between 0–2856 m, on average 198.8 (± SD 192.3) m for females and 442.5 (± SD 448.2) m for males. We extracted individual movement bursts, i.e. continuous periods of movement that are interrupted by periods of inactivity, from the above-mentioned nightly tracking periods. There is a possibility that not all the foraging times were detected, and thus nightly movement distances may include few periods of foraging in trees. We measured home-range size using a 100 % minimum convex polygon (MCP) to enable comparisons to earlier studies on flying squirrel space use [36, 38]. The mean MCP home-range size was 6.8 (± SD 4.9) ha for females and 65.0 (± SD 40.4) ha for males. Home-range sizes and lengths of the movements were calculated with the R packages adehabitatHR and adehabitatLT [45].

### Statistical analyses based on generalized linear mixed models

We applied log-normal models to assess factors influencing flying squirrel movement patterns as measured by three response variables (Table 1): the lengths and the speeds (length divided by duration) of individual movement bursts, and the nightly total movement distances, i.e. the lengths of the entire movement trajectories during one night. As explanatory variables for the length and speed of the movement burst, we used the sex of the individual, the month of the year (from March to September; categorical variable), the proportions of the habitat classes H1–H3 within a 25-m buffer along each movement burst, and interactions between sex and month, and sex and habitat proportions. The 25-m radius was chosen to describe the fine-scale habitat along movement paths. To control for the possibility that longer movement bursts contained a larger proportion of short-term periods of inactivity, we included the log-transformed duration of the movement burst as an explanatory variable when modelling the speed of the burst. Explanatory variables for the nightly moved distance were sex, month, proportions of the habitats H1–H3 within the home range of the individual, and interactions between sex and month, and sex and habitat proportions. Variation in observation effort was controlled for by adding the log-transformed total time of the tracking period as an explanatory variable. As we had repeated measurements from the same individuals, we included individual as a random effect of the intercept in the models. Unsuitable habitats (H4) and water bodies (H5) are known to be avoided by flying squirrels, and as their proportions within buffered movement bursts and home ranges were small we excluded them from analyses. We checked for collinearity of variables with pairwise correlation coefficients (max. -0.67 between H1 and H3) and with variance inflation factors (max. 7.1) [46]. We used the Akaike’s information criterion- approach (AIC) for selecting the best supported models [47] (but see [48] for a discussion of the limitations of AIC). AIC-values corrected for sample sizes (AIC_*c*_) were used for model comparison, and all the models that gave reasonable support (with ΔAIC_*c*_ < 2), were included in model averaging. Effects of variables were examined with the help of variate weights and 95 % confidence intervals [49, 50].

**Table 1:** Explanatory variables included in the GLMM analyses (x denotes inclusion of the variable in the model)

Model	Sex	HR	H(HR)	H(burst)	M	D(burst)	D(night)	E(sites)	Id	Sex*HR	Sex*H(HR)	Sex*H(burst)	Sex*M
A: length of burst	x			x	x				x			x	x
B: speed of burst	x			x	x	x			x			x	x
C: nightly distance	x		x		x		x		x		x		x
D: number of nests	x	x	x					x		x	x		

Sex models the effect of the individual being a male, and thus female was set as the reference level. HR refers to home-range size (100 % MCP). H(HR) and H(burst) refer to the proportions of different habitat types within the home range and within a 25 m radius buffer along the movement burst, respectively. Used habitat types were H1–H3 (see Methods). Month (M) from March to September was included as a categorical variable, with March set as the reference level. D(burst) and D(night) refer to the log-transformed durations of the burst and the nightly tracking period, respectively, whereas E(sites) refers to the log-transformed number of days during which the nest site of the individual was recorded. Id refers to individual that was used as a random effect to control for repeated measurements. In models A–C we log-transformed the response variables and applied a normal model, whereas in model D we applied Poisson regression with the log link function

We applied Poisson regression with the log-link function to examine which factors influence the number of nest sites within the home range, with sex, home-range size and proportions of habitat classes and their interactions with sex as explanatory variables. Observation effort was controlled for by including the log-transformed number of days during which the nest site of the individual was monitored as an explanatory variable (Table 1). Sufficiency of sampling was assessed by creating a rarefaction curve using R package vegan [51, 52]. Here, rarefaction describes the change in number of nests with increasing observation effort (Additional file 2). Number of nests had a tendency to level off with our sampling intensity, which suggests we did not miss a high fraction of nest sites.

### Realized habitat use versus habitat availability within the home range

We examined flying squirrel habitat preferences by running a compositional analysis, which is suited for studying habitat use with data on several individuals when habitat is classified into discrete categories [53, 54] (for alternative methods, see [55, 56]). To quantify habitat availability regarding the proportions of the habitat types H1–H4 within the home ranges, the study area was rasterized to a resolution of 25 m, and the proportions of cells belonging to different habitat types within the 100 % MCPs were computed. To quantify habitat usage, we calculated the proportions of habitat types at the recorded locations. Proportions of used habitats were compared to proportions of available habitats following Aebischer et al. (1993) and by using the R package adehabitatHS [45]. Significance of habitat selection and ranking were tested with randomization tests and Wilks Lambda (Λ) [57], using the *p*-value 0.05 as a threshold. To avoid singular cases, we replaced 0-values for habitat use with the value of 0.01. For missing values created by zero habitat availability, we replaced the log-transformed ratio between used and available habitat by the mean value of other individuals [53]. The effect of sex was examined by running the compositional analysis separately for the sexes and by comparing the results. All the above-mentioned statistical analyses were performed with R version 3.0.2 [58].

### Movements among the network of daytime nest sites

We used a Markov chain model to study the effect of habitat connectivity on the probability of switching between nest sites. We constructed the chronological sequence of daytime nest sites used by each individual, and modeled the transitions between the sites. The Markovian assumption implies that the state of the system *z*(*t*), i.e. a vector of length *n* including the probabilities of the individual being in each of the *n* nest sites at day *t*, depends only on the state of the system in the previous day, *z*(*t* − 1). The state *z*(*t*) evolves as:

$$ \mathbf{z}\left(t+\Delta t\right)=\mathbf{z}(t){\mathbf{P}}^{\Delta t} $$

where *Δt* is the number of days between the observations, and the element *P*_*ij*_ of the transition matrix **P** is the probability that the individual will move to nest site *j* if it currently is in nest site *i*. The diagonal terms *P*_*ii*_ model the probabilities that the individual stays in the same site. We modelled the transition matrix **P** through Multinomial regression. We denoted the linear predictor related to movement probability from site *i* to *j* by *L*_*ij*_, and modelled the probability of switching from site *i* to site *j* as ${P}_{ij}={e}^{L_{ij}}/{\displaystyle \sum_j}{e}^{L_{ij}},$ where the denominator is a scaling constant ensuring that ${\displaystyle \sum_j}{P}_{ij}=1.$ As predictors for the linear predictor in the transition matrix **P** we included the identity matrix **I** to capture the probability of staying in the same site, the distance matrix **D** specifying the distance (transformed as log(distance + 1 meter)) between the sites; and the binary connectivity matrix *C*, specifying whether two sites were connected by habitat (0 = not connected, 1 = connected). Denoting by *β*_*I*_, *β*_*D*_ and *β*_*C*_ the regression parameters related to staying in the same nest, the effect of distance, and the effect of connectivity, the model thus becomes:

$$ \mathbf{L} = {\beta}_I\mathbf{I}+\left(1-\mathbf{I}\right)\left({\beta}_D\mathbf{D}+{\beta}_C\mathbf{C}\right) $$

where the term (1 − **I**) implies that the effects of distance and connectivity are modelled conditional upon the individual switching to another nesting site. We denote the individual by the subscript *l* = 1, …, *n*, where *n* is the number of individuals. We assumed individual-specific parameters, which we combine to the vector *β*_*l*_ = (*β*_*I*,*l*_, *β*_*D*,*l*_, *β*_*C*,*l*_)^*T*^. We used a multivariate normal distribution to model variation among individuals and thus assume ***β***_*l*_ ~ *N*(***μ***_*l*_, **Σ**). Here the mean response *μ*_*l*_ (vector of length 3) is assumed to depend on the sex of the individual, and so *μ*_*l*_ = *α*_0_ + *α*_1_*s*, where *α*_0_ is the mean response and *α*_1_ is the effect of the sex *s* of the individual (-1 = females, 1 = males). The matrix **Σ** (a 3 × 3 variance-covariance matrix) models variation among individuals not captured by the effect of sex.

We defined the twelve different connectivity matrices (C1–C6 and C1g–C6g) among the pairs of nest sites to test what kind of connectivity measure best explains flying squirrel movements. A pair of nest sites was considered to be connected if it was possible to draw a route from one site to the other site so that C1) the route followed a straight line and was entirely located within suitable habitat within the home range; C2) the same as C1, but the route also included movement habitat; C3) and C4): the same as C1) and C2), but the route was not required to be a straight line; C5) and C6): the same as C3) and C4), but the route was not required to be located entirely within the home range. We also considered variants of these six connectivity measures for which two sites were considered connected even if the route included gaps (i.e. areas not classified as suitable habitat or movement habitat) of max 50 m wide, and denote these by C1g–C6g (for example of the routes between nest sites see Additional file 3). To assess the effects of different predictor variables, we parameterized the model with distance only (1 model), connectivity only (12 models, one for each connectivity measure), and distance and connectivity (12 models, one for each connectivity measure).

We fitted the model within a Bayesian framework because it allowed us to account for joint parameter uncertainty in the non-linear model. We assumed uniform priors for the regression parameters *α*, and an inverse-Wishart prior with mean set to the identity matrix and degrees of freedom to 4. We chose to use the non-informative prior for the regression coefficients due to lack of prior information. The choice of the Inverse-Wishart prior for Sigma was made for computational convenience, as it is the conjugate prior for variance covariance matrices. To reflect the lack of prior information, the degrees of freedom parameter was set to the minimal value that makes the distribution proper [59]. We sampled the posterior with a Markov chain Monte Carlo (MCMC) approach. We used a random walk Metropolis–Hastings algorithm with a multivariate normal proposal distribution for the *β* -parameters. We used 25,000 iterations, of which the first 5000 were considered as burn-in iterations during which we adaptively scaled the proposal distribution to achieve an acceptance ratio of 0.23 (see [60] for more details). As we defined conjugate priors for *α* and **Σ**, these parameters were sampled from their full conditionals. We checked for convergence and mixing through inspection of the trace plots and by comparing multiple chains initiated from different initial values. We used the deviance information criterion (DIC) to compare the models [61].

## Results

### Effects of small-scale landscape composition on movement patterns

**Fig 2:**
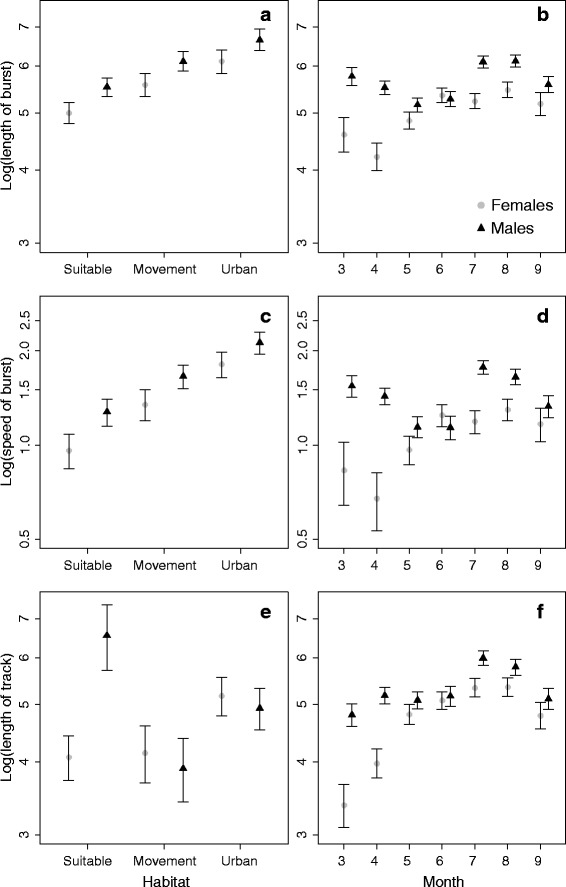
Lengths (**a**, **b**) and speeds (**c**, **d**) of movement bursts, and lengths of nightly tracks (**e**, **f**) predicted by the GLMMs. Dots show the predicted values separately for female and male flying squirrels assuming that all habitat would consist of one particular type (*left panels*), and as a function of month from March to September (*right panels*). Whiskers denote standard errors. The response variables were log-transformed and explanatory variables that are not varied in the figure were set to their mean values

Average proportions of habitat classes within buffered movement bursts were 56.8 (± SE over individuals 6.7 %) and 51.2 (± SE 4.7 %) for suitable habitat, 21.1 (± SE 6.3 %) and 26.2 (± SE 4.6 %) for movement habitat, and 17.3 (± SE 5.1 %) and 14.9 (± SE 3.7 %) for urban habitat, for females and males, respectively. Movement bursts were shorter for females (mean 184.1 ± SE 13.2 m) than for males (mean 453.0 ± SE 26.6 m), and they were longer if their vicinity (radius of 25 m) included urban habitat (Table 2, model A; Fig. 2a). Lengths of the movement bursts of females increased nearly continuously over months whereas males made the longest movements both in spring time (March-April) and in late summer (in July and August)(Fig. 2b).

**Table 2:** Results of model averaging across the highest ranked (ΔAIC_*c*_ < 2) GLMMs for each response variable (from A to D)

Model	A: length of burst			B: speed of burst			C: length of nightly track			D: number of nest sites		
	Estimate	SE	*w*	Estimate	SE	*w*	Estimate	SE	*w*	Estimate	SE	*w*
Parameters												
Intercept	4.265	0.449		0.897	0.341		−8.484	1.135		−2.441	0.856	
Sex^a^						1.00			1.00			
Males	1.083	0.372	1.00	0.640	0.231		1.573	0.486				
Month^a^						1.00			1.00			
April	−0.390	0.362	1.00	−0.150	0.222		0.565	0.346				
May	0.201	0.331		0.099	0.203		1.390	0.330				
June	0.717	0.327		0.383	0.201		1.652	0.325				
July	0.584	0.335		0.312	0.206		1.908	0.336				
August	0.774	0.336		0.381	0.207		1.931	0.339				
September	0.476	0.377		0.257	0.231		1.422	0.374				
H1 (suitable)	0.726	0.484	0.41	−0.038	0.290	0.16	0.057	0.820	0.27	−0.453	0.552	0.22
H2 (movement)	0.700	0.529	0.78	0.293	0.192	0.71	−0.916	0.492	0.63	0.425	0.467	0.24
H3 (urban)	1.295	0.514	1.00	0.748	0.241	1.00	0.852	0.619	0.56			
Sex^a^*Month^a^			1.00			1.00			1.00			
Males: April	0.191	0.425		0.075	0.261		−0.187	0.427				
Males: May	−0.819	0.400		−0.506	0.245		−1.093	0.420				
Males: June	−1.114	0.407		−0.719	0.250		−1.284	0.429				
Males: July	−0.239	0.410		−0.077	0.252		−0.710	0.432				
Males: August	−0.400	0.413		−0.278	0.254		−0.949	0.435				
Males: September	−0.736	0.454		−0.535	0.279		−1.113	0.477				
Sex^a^*Habitat												
Males: H1							1.813	0.965	0.14			
Males: H2	0.307	0.500	0.23	0.184	0.309	0.07						
Males: H3				0.390	0.353	0.21	−1.540	0.704	0.56			
HR										0.239	0.054	1.00
D(burst)				−0.095	0.071	0.35						
D(night)							1.375	0.125	1.00			
E(sites)										0.388	0.156	1.00

Model averaged parameter estimate, associated standard error (SE) and variate weight (*w*) is provided for each explanatory variable that was chosen among the highest ranked models. Bolded value indicates that 95 % confidence intervals of a parameter estimate does not include zero

^a^Denotes categorical variable where the effect of the first category (Sex: Females or Month: March) was set at the reference level. The variables are defined in the methods section and the complete list of models used in model averaging is provided in Additional file 4

Average movement speeds were 2.5 (± SD 2.0) m/min for females and 4.1 (± SD 3.8) m/min for males. Movement speed increased with the proportion of urban habitat and was lower for females than for males (Table 2, model B; Fig. 2c). Variation in movement speed with month showed patterns similar to that of burst length (Fig. 2d): the movement speed of females peaked in August whereas males had two peaks of higher activity (March and July). Duration of the burst had no effect on the movement speed.

### Effect of home-range habitat composition on nightly moved distance and number of distinct nest sites

Average proportions of habitat types within home ranges were 43.6 (± SE over individuals 7.2 %) and 23.7 (± SE 2.4 %) for suitable habitat, 21.0 (± SE 5.9 %) and 24.8 (± SE 3.4 %) for movement habitat, and 28.6 (± SE 6.4 %) and 30.7 (± SE 4.5 %) for urban habitat, for females and males, respectively. Total distance moved during nightly tracking periods were affected by the duration of the tracking period, sex and by habitat composition within the individual’s home range (Table 2, model C). As expected, the longer the duration of the nightly tracking period, the longer the total distance moved. The presence of urban habitat within the home range affected distances travelled by both sexes. However, while it increased the distances travelled by females, this was not the case for males (Fig. 2e). Nightly moved distances were the greatest for both sexes in July and August, and the lowest in March (Fig. 2f).

**Fig. 3:**
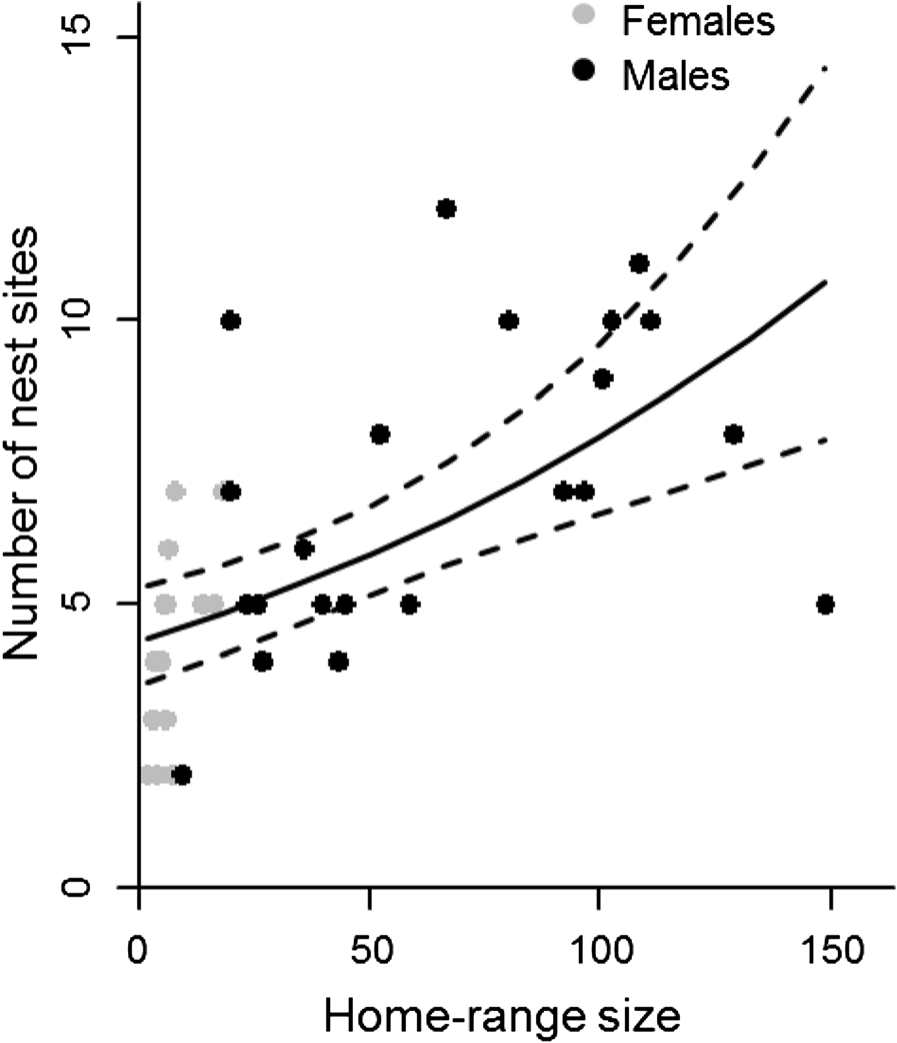
The number of distinct nest sites increases as a function of home-range size. The curves show the prediction of the Poisson regression model. The dashed lines denote 95 % confidence intervals. Dots represent the observed number of nests for females (*n* = 17) and males (*n* = 22). The explanatory variable that is not varied (number of days the nest sites were recorded) was set to its mean value

We identified a total of 232 nest sites for all the individuals (note that this number included same nests that were used by different individuals). Females had on average 4.2 (± SD 1.6) nest sites and males on average 7.0 (± SD 2.7) nest sites. Of the total nest sites recorded 48 % were cavities, 41 % were twig nests, 8 % were nest boxes and 2 % were nests in buildings. Most of the nest sites were located in mature spruce-dominated forests, i.e., in the suitable breeding habitat type. Of the remaining nests, 20 % were in movement habitat, 10 % in urban habitat and 3 % in unsuitable habitat. As expected, the number of nests sites was positively associated with observation effort, i.e., the number of days a nest site was recorded for each individual (Table 2, model D). The number of distinct nest sites increased with home-range size similarly for both sexes, even if home-range sizes of males were much greater than those of females (Fig. 3). Neither the proportions of habitats nor their interactions with sex had significant effects on the number of nest sites (Table 2).

### Habitat use during movements versus habitat availability within home range

During movement bursts all flying squirrels used suitable habitat significantly more than other habitat types (Table 3). Other habitat types were used in the following order for females: *movement habitat > urban habitat > unsuitable habitat*, whereas for males the order was *movement habitat > unsuitable habitat > urban habitat* (Table 3). According to randomization tests of the analysis, habitat selection was significant for the data pooled over all individuals (Λ = 0.297, *p* = 0.001), and also separately for females (Λ = 0.240, *p* = 0.003) and males (Λ = 0.321, *p* = 0.003).

**Table 3:** Ranking matrices of habitats used during movements versus habitats available within the home range

Habitat type	Suitable	Movement	Unsuitable	Urban	Rank
1) All (*N* = 39)					
Suitable	0.000	**1.603** ^a^	**3.829** ^a^	**3.252** ^a^	1
Movement	−1.603	0.000	**2.113** ^a^	**1.580** ^a^	2
Unsuitable	−3.829	−2.113	0.000	**0.090**	3
Urban	−3.252	−1.580	−0.090	0.000	4
2) Females (*n* = 17)					
Suitable	0.000	**0.630** ^a^	**3.181** ^a^	**2.002** ^a^	1
Movement	−0.630	0.000	**2.629** ^a^	**1.464** ^a^	2
Unsuitable	−3.181	−2.629	0.000	−0.445	4
Urban	−2.002	−1.464	**0.445**	0.000	3
3) Males (*n* = 22)					
Suitable	0.000	**1.418** ^a^	**3.483** ^a^	**3.594** ^a^	1
Movement	−1.418	0.000	2.166	**2.434** ^a^	2
Unsuitable	−3.483	−2.166	0.000	**0.666**	3
Urban	−3.594	−2.434	−0.666	0.000	4

The results are shown 1) for all individuals, 2) for females only, and 3) for males only. Proportions of habitats used during movement bursts were compared to proportions of habitats within the home range (100 % MCP) by compositional analysis. Ranking matrix shows the mean differences between log-ratios of used and available habitat types. When habitat in a row is used more than habitat in a column, the value is bolded, and additionally ^a^ denotes that the difference in use significant at the 0.05 level. Last column shows the ranking of habitat use (1 = most used, 4 = least used)

Effect of distance and connectivity on switching probability between the daily nest sites

We obtained data on nest-site switching for 19 females and 22 males. Distances between nest sites were much shorter for females (maximum ca 600 m) than for males (maximum ca 2000 m). Maximum distance between nest sites connected continuously by a straight line and by suitable habitat (C1) was about 300 m for both sexes.

**Fig. 4:**
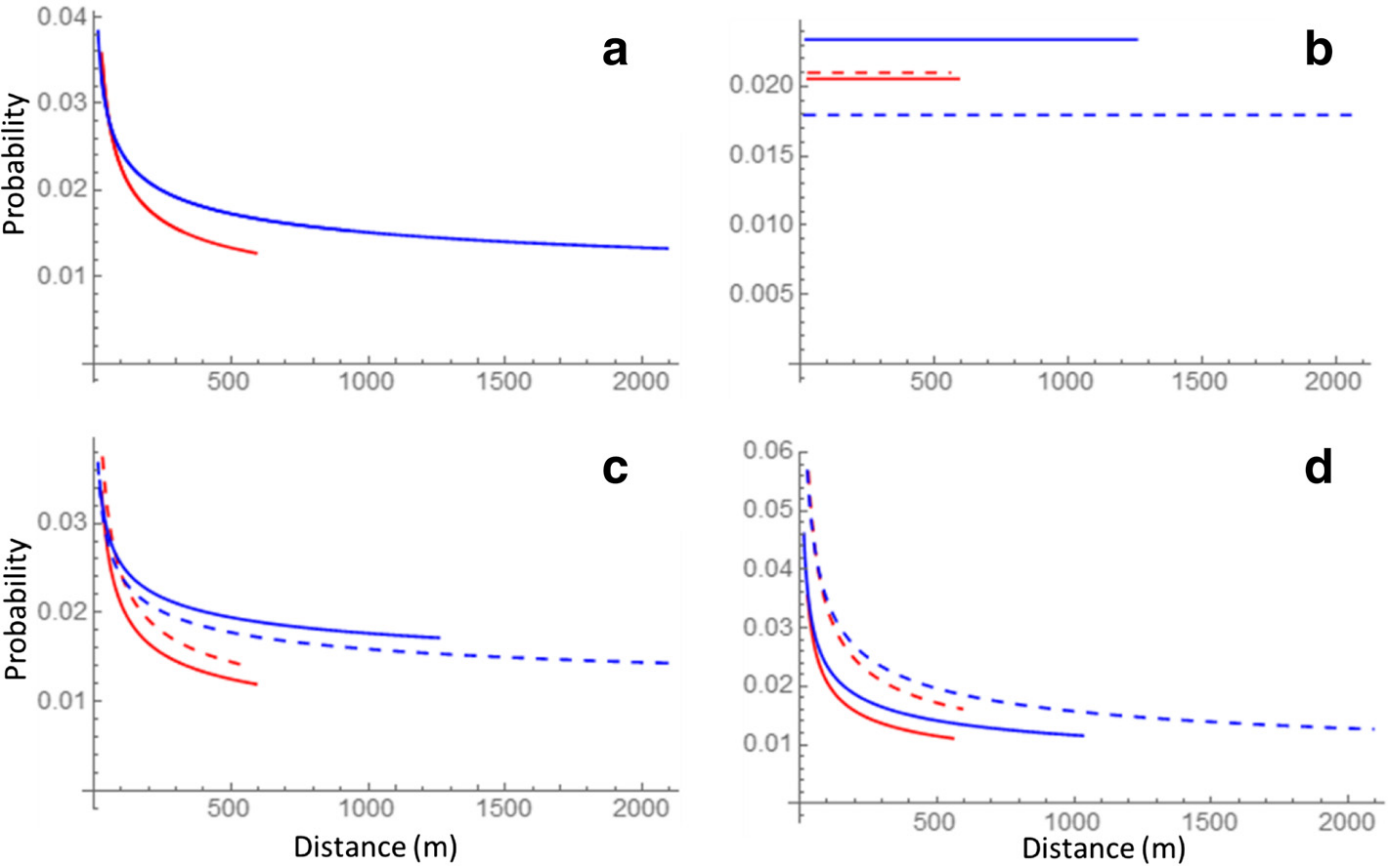
The influence of sex, distance and connectivity on nest-switching probability. Females are shown in red and males in blue. Continuous lines correspond to connected nests (or all nests in the distance only model) and dashed lines to unconnected nests. The lines are drawn to span their full range in the data. The predictions are made for a male with 7 nests (median number of nests for males) and a female with 4 nests (median number of nests for females). We kept the distances and connectivities to other nests as they were in the real data, but varied the properties of the target nest. The panels show the predictions of the models with **a** distance only, **b** connectivity only, by C5g (model 11), **c** distance and connectivity by C5g (model 11), **d** distance and connectivity by C2g (model 8)

The model with distance only (DIC = 2214) showed that for both sexes, the switching probability decreased with distance (Fig. 4a). The best supported models with connectivity only (DIC = 2212 for model C5g, ΔDIC ≥ 3 for other connectivity measures) showed that the switching probability is higher among connected sites for males, but is not influenced by connectivity for females (Fig. 4b). As connected nests were on average closer to each other than unconnected nests, in particular for males, this result is consistent with the negative influence of distance. The best supported models with both distance and connectivity included (DIC = 2209 for model C5g, ΔDIC = 1 for model C2g, ΔDIC ≥ 3 for other models) also showed that distance has a negative influence, but they gave contradictory results for the effect of connectivity. Both connectivity measures had a negative effect on switching probability for females, but C5g (i.e., nest sites are connected by suitable forests, also routes outside home-range boundaries and gaps allowed) increased and C2g (i.e., connected by forested habitat by a straight line, but gaps allowed) decreased switching probability for males (Fig. 4c and d, respectively). Thus, there was no clear evidence for connectivity influencing the switching probabilities after accounting for the effect of the distance.

## Discussion

We found that flying squirrels responded to increasing amounts of urban habitat along their movement paths by moving faster and longer distances. In general, various movement patterns of forest-dwelling rodents have been observed in the landscape matrix: animals can move faster and more directly, slower and more tortuously, or movements can be interrupted by short stops [62–64]. The observed faster movements as a response to urban habitat suggest that movement mode was likely straight or nearly straight and individuals headed for specific locations. When urban habitat does not provide important resources for the species, it is more efficient to move quickly through the less suitable landscape [65]. Animals may also try to minimize time spent in unsuitable areas, for example, when the habitat is considered more risky [66]. Since predator pressure can alter space use and habitat selection of individuals [67], and it is yet unknown how flying squirrels perceive the predation risk in modified habitats or how large the mortality risk while moving is, this subject requires more investigation. Isolation and increased distances between suitable patches in fragmented and less-forested landscapes have increased dispersal distances of the flying squirrel and the white-tailed deer (*Odocoileus virginianus*) [68, 69]. Hence, our results on longer movement paths over urbanized landscape matrix could be related fragmentation of the suitable forests by urban land use when individuals need to move further to reach suitable foraging patches. However, there was some heterogeneity within the urban habitat class, such as areas with trees, and thus, more fine-scale habitat features could also have directed the flying squirrel movements.

Earlier studies indicate that moving through urban landscape can be impeded by the urbanization of the matrix since sugar gliders (*Petaurus breviceps*) move less in the more urbanized matrix [22] and squirrel gliders (*Petaurus norfolcensis*) are using larger core areas in continuous forests than in forest fragments [70]. Results of flying squirrel males show that nightly movement distances were longer in home ranges that contained a lot of suitable forests than in home ranges that contained a lot of urban habitat types. Male flying squirrels have large territories, can traverse throughout their home range within one night, and regularly visit territories of several females [36]. However, when a home range involves a large fraction of inhospitable habitat, individual may spend several days in one part of the home range before crossing through the matrix to reach another core area due to costs of moving through matrix. In contrast, the availability of suitable habitat had less influence on the nightly moved distances of female flying squirrels, whose home ranges are smaller and often confined within one suitable forest patch [36]. In our case, home ranges of females included more mature spruce-dominated forests that are suitable for breeding than home ranges of males (44 % vs. 24 %). Thus, females have most probably chosen forest patches that are big enough for breeding and raising the young, and as they are territorial they virtually never move outside the home-range they are occupying.

Modification of forests by human land use may affect the availability of nest sites and spacing behaviour of a species. Observations of flying squirrels showed that the number of distinct nest sites was greater in large home ranges. Flying squirrels can use twig nests or buildings, but good nesting cavities are important, especially for breeding females, and lack of cavities could hinder their breeding and rearing of young, lowering overall survival of the young. In addition, individuals with greater amount of nests might benefit by being able to switch to alternative nest when ectoparasite load or predation pressure on the current nest changes [71, 72]. Thus, the availability of nest sites may influence the space use of the species affecting both the shape and size of the home range. For other forest-dwelling animals, the number of nests has either decreased or increased with human disturbance, or nests have become concentrated in less fragmented areas [73–75]. For instance, the northern goshawk (*Accipiter gentilis*) also seems to suffer from forest harvesting that reduces the area of mature forests and number of alternative nest sites [76]. We found no significant effect of the habitat composition within a home range on the number of nest sites, but its potential association with the availability of nest types should be further investigated.

Our results indicate that extensive space-use by gliding squirrels, especially males, also leads them to utilize unsuitable areas, such as sparsely forested habitat types. We observed differences in habitat preferences between the sexes, as males were using more unsuitable habitats than females. This might be because animals need to occasionally cross such habitats in order to reach another part of their home range. There might also be some fine-scale habitat features such as single large trees on clear cut areas or sapling stands that can be used by moving individuals. The use of urban habitats could indicate that individual home ranges are located at the edge of forests, for example near old residential areas at low-contrast edges that could provide suitable habitat and food resources due to increased productivity at the edges [25, 77]. Therefore, we acknowledge that the effects on differential urban habitat types on the movement responses of this species should still be clarified.

Temporal variation in the availability and quality of food resources as well as breeding activities may affect the seasonal patterns in movements. For example, red squirrels exploit larger areas if food is scarce [78], but restrict their movements and only defend high-quality core areas if food is abundant [79]. Our results showed that flying squirrel movements varied with month and were sex-dependent. Forests at our study site are herb-rich and contain plenty of deciduous trees where flying squirrels can forage upon food items that can be accessed almost year-round (e.g., catkins in spring and leaves in summer), although there is seasonal variation in availability of the different food types. Thus, long distances moved by males during early spring are most likely caused by the mating season during which they search for females [38]. Although leaf food is available since mid-May, females move little because they have to stay close to nest to take care of the juveniles, while the peak in movements at the end of summer could be related to individuals preparing for the winter by spending the reasonably short nights for foraging [38]. Therefore, it is unlikely that food availability has a major influence on flying squirrel movement patterns at our study area where food is not a limiting factor.

In line with our hypothesis, switching probability was high between nest sites that located close to each other. Moving to a nearby nest could also be easier because structurally connected nest sites are more often closer to each other than unconnected ones. After accounting for the effect of distance, our models gave contradictory results on the effect of connectivity depending on the measure used. Connectivity had a different influence on males: when nests were connected allowing routes outside home-range boundaries, switching probability for males became higher, but when connection was a straight line switching to nest was smaller. We attribute this inconsistency to the difficulty of measuring connectivity in a way that is relevant to the animals [80], or to confounding factors that were not measured such as differential preferences for the different nest types. For example, cavities might be preferred over twig nests, or some nest sites may provide more shelter than others [73, 75]. Additionally, large-scale habitat selection may influence lower-level patterns, for instance, female flying squirrels may have already selected their territories to be in a continuously forested area large enough, and because of this their nest sites lie within one forest fragment [81].

The effect of connectivity on animal movements in fragmented landscapes was shown by earlier studies on forest dependent species, although it has not been related to nest-site switching. For instance, the probability of returning home has been greater in connected landscapes for northern flying squirrels and ringtail possums (*Hemibelideus lemuroides*), and presence of gaps has increased returning time for forest birds [82–84]. In our case, we conclude that the presence of continuous forest corridors is not a necessary condition for flying squirrels changing their nest sites. Indeed, male flying squirrels regularly cross gaps up to 50 meters in forest cover [37, 85]. Hence, it is important to further investigate if management of the interspersed matrix could be used to increase connectivity and secure movements between separate habitat patches [22].

## Conclusions

On the one hand, our findings show that flying squirrels are able to inhabit urban areas and to change their behaviour according to habitat type and landscape structure. Since the flying squirrel population decline is ongoing in forested areas in Finland, protecting the species in urban environments becomes increasingly important and is an interesting possibility. On the other hand, our results highlight the importance of mature forests. We propose that for conserving the species in urbanized areas, enough suitable mature forest for breeding should be maintained at the home-range scale, whereas connectivity between nearby forest patches should be ensured by providing suitable habitat for movements.

Our results indicate that landscape composition can affect the movements of a forest-dwelling species differently on a small scale compared to the larger home-range scale. Faster movements through urbanized habitats indicates that these habitats are less favoured. Increasing amount of urban habitat within home range decreased distances moved by males, suggesting that movements of the more mobile sex could also be hampered by urbanization of the landscape. The importance of forested connections remains unclear and it seems that measuring and maintaining connectivity in a species-specific way is difficult in human-modified landscapes.

Habitat selection and home-range establishment of individuals can ultimately influence their survival and life-time reproductive success, which can have consequences at the whole population level [86, 87]. Since destruction of native habitats is ongoing, protection of many species has to be done in modified landscapes in the future. In order to estimate how well this can succeed for gliding and arboreal squirrels, we propose that the link of habitat use to cost and risks of moving in fragmented landscapes should be studied next.

### Ethics approval

The procedure of this study was made in accordance with current laws in Finland and under the license from the Centre for Economic Development, Transport and the Environment (permit number: POSELY/608 /07.01/2010).

### Consent for publication

Not applicable.

## Additional files

Additional file 1:
**Description of the yearly data collected during field work.** Numbers of followed individuals and tracking periods, and means and standard errors (SE) of moved distances, tracking times, home-range sizes (100 % Minimum convex polygons) and number of locations used for home range estimate are shown separately for females and males. (DOCX 14 kb) Additional file 2:
**Change in the number of nest sites with the increasing observation effort.** Solid curve shows the cumulative number of nest sites with the growing number of observations when observations are added in order they exist in the data. Dashed curve is generated by a rarefaction method, which finds the mean by sampling among all individuals (Colwell et al. 2012). Here, it describes that number of nests found tends to level off with our sampling intensity. Observation effort denotes a number of days nest site was monitored for an individual. (DOCX 37 kb) Additional file 3:
**Example of home-range habitat composition, and locations and connectivity of distinct nest sites of a female Siberian flying squirrel.** A female home range by 100 % minimum convex polygon (MCP) is delineated by the black dashed line and numbered stars denote for the distinct nest sites. Different connectivity measures (see methods for details) are shown by blue arrowed lines. Individual could move from nest 1 to nest 2 by a straight line, or tortuously inside or outside home-range boundary, but in all cases the track would also comprise of movement habitat (connectivity measures C2, C4 and C6). However, if moving between nest sites 1, 3 and 4, all movements fall within the suitable habitat (connectivity measures C1, C3 and C5). In order to move from nest 6 to 7, female could move directly via suitable forest or taking detour, but it had to cross a gap in tree cover (connectivity measures C1g and C2g). (DOCX 862 kb) Additional file 4:
**Results of model selection and comparison by Akaike’s Information Criteria (AIC).** For each response variable (from A to D) a list of highest ranked models (within ΔAIC_*c*_ < 2) and their explanatory variables are shown. For every model the difference of AIC_*c*_ (AIC corrected for small sample sizes) from the best approximated model (lowest AIC) and Akaike weight (w_*i*_) is provided. (DOCX 13 kb)

## Abbreviations

AIC: Akaike’s information criterion
DIC: Deviance information criterion
GLMM: Generalized linear mixed model
MCMC: Markov chain Monte Carlo
MCP: Minimum convex polygon
