# Erratum to: Home-range use patterns and movements of the Siberian flying squirrel in urban forests: effects of habitat composition and connectivity

**DOI:** 10.1186/s40462-016-0076-7

**Published:** 2016-04-14

**Authors:** Sanna Mäkeläinen, Henrik J. de Knegt, Otso Ovaskainen, Ilpo K. Hanski

**Affiliations:** Finnish Museum of Natural History LUOMUS, University of Helsinki, P. O. Box 17 (P. Rautatiekatu 13), Helsinki, FI-00014 Finland; Department of Biosciences, University of Helsinki, P. O. Box 65 (Viikinkaari 1), Helsinki, FI-00014 Finland; Current address: Resource Ecology Group, Wageningen University, Droevendaalsesteeg 3a, Wageningen, 6708 PB The Netherlands

After publication of this study [1], we noticed that Fig. 1 was not correctly processed during copyediting. The original version of this article was corrected. The publisher apologizes for any inconvenience caused. Please see the corrected Fig. 1 below:

**Fig. 1:**
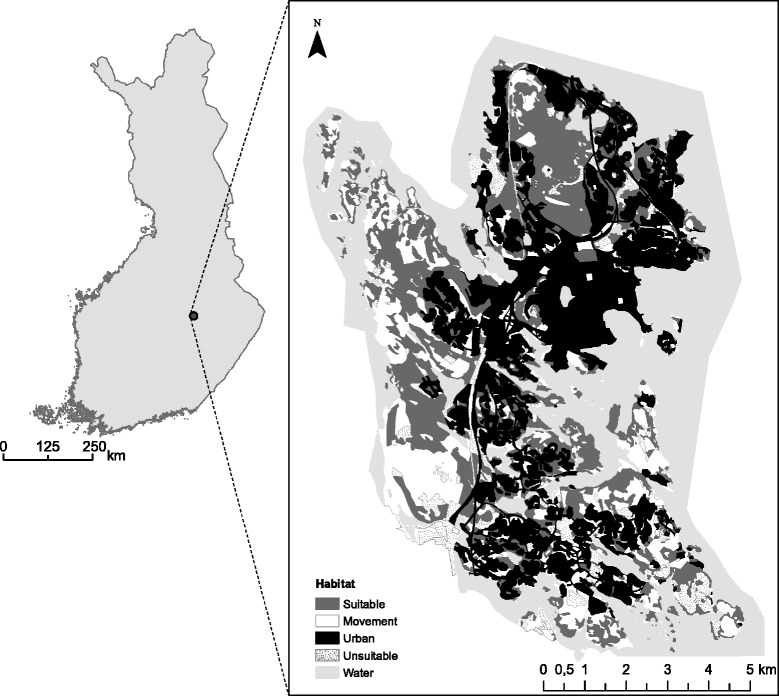
Map and habitat classification of the study area in Kuopio, Eastern Finland. Habitats are classified by their suitability for the flying squirrel. Suitable habitat (H1) denotes mature spruce-dominated forests. Movement habitat (H2) consists of forests that are over 10 m in height. Urban habitat (H3) consist of residential areas, roads or other habitats dominated by human land use. Clear cut areas, fields and sapling stands are combined to unsuitable habitat (H4). Water bodies (H5) are not utilized by the species and may form barriers for movementsᅟ

## References

[1] Mäkeläinen S, de Knegt HJ, Ovaskainen O, Hanski IK (2016). Home-range use patterns and movements of the Siberian flying squirrel in urban forests: Effects of habitat composition and connectivity. Mov Ecol.

