# Supplementary material for: Home-range use patterns and movements of the Siberian flying squirrel in urban forests: Effects of habitat composition and connectivity
Source: Mov Ecol. 2016 Feb 17;4:5. doi: 10.1186/s40462-016-0071-z (PMC4758174; doi:10.1186/s40462-016-0071-z)
Supplement: Additional file 1: — Description of the yearly data collected during field work. Numbers of followed individuals and tracking periods, and means and standard errors (SE) of moved distances, tracking times, home-range sizes (100 % Minimum convex polygons) and number of locations used for home range estimate are shown separately for females and males. (DOCX 14 kb) [file 40462_2016_71_MOESM1_ESM.docx]

| **Year** | **Sex** | **Individuals** | **Tracking periods** | **Moved distances (m)** | **Tracking times (min)** | **Home-range size (ha)** | **Locations** |
| --- | --- | --- | --- | --- | --- | --- | --- |
| 2008 | females | 4 | 67 | 204.4 (± 22.9) | 86.7 (± 4.4) | 9.7 (± 0.7) | 107 (± 5.2) |
|  | males | 1 | 19 | 369.0 (± 50.7) | 89.6 (± 5.6) | 8.8 (± 0.0) | 133 (± 0.0) |
| 2009 | females | 4 | 63 | 132.9 (± 17.6) | 91.9 (± 5.4) | 6.0 (± 0.7) | 111 (± 33.0) |
|  | males | 5 | 143 | 406.6 (± 39.9) | 110.6 (± 3.3) | 72.3 (± 3.4) | 228 (± 32.3) |
| 2010 | females | 4 | 61 | 269.2 (± 33.4) | 100.3 (± 5.6) | 9.3 (± 0.7) | 119 (± 9.1) |
|  | males | 7 | 57 | 502.6 (± 67.5) | 113.5 (± 4.0) | 79.7 (± 4.8) | 83 (± 25.9) |
| 2011 | females | 2 | 15 | 184.2 (± 30.5) | 111.5 (± 10.8) | 4.5 (± 0.4) | 56 (± 13.3) |
|  | males | 8 | 68 | 429.3 (± 46.9) | 101.8 (± 4.0) | 76.7 (± 4.8) | 73 (± 7.7) |
| 2012 | females | 6 | 38 | 198.3 (± 26.8) | 96.8 (± 4.7) | 4.6 (± 0.3) | 44 (± 5.7) |
|  | males | 3 | 20 | 319.6 (± 67.2) | 97.9 (± 5.8) | 44.6 (± 7.8) | 45 (± 14.4) |
| **Total** |  | **44** | **551** |  |  |  |  |
