# Supplementary material for: Home-range use patterns and movements of the Siberian flying squirrel in urban forests: Effects of habitat composition and connectivity
Source: Mov Ecol. 2016 Feb 17;4:5. doi: 10.1186/s40462-016-0071-z (PMC4758174; doi:10.1186/s40462-016-0071-z)
Supplement: Additional file 2: — Change in the number of nest sites with the increasing observation effort. Solid curve shows the cumulative number of nest sites with the growing number of observations when observations are added in order they exist in the data. Dashed curve is generated by a rarefaction method, which finds the mean by sampling among all individuals (Colwell et al. 2012). Here, it describes that number of nests found tends to level off with our sampling intensity. Observation effort denotes a number of days nest site was monitored for an individual. (DOCX 37 kb) [file 40462_2016_71_MOESM2_ESM.docx]

10

20

30

40

50

60

0

2

4

6

8

10

12

Observation effort (days)

Cumulative number of nest sites
