# Supplementary figures and images for: Home-range use patterns and movements of the Siberian flying squirrel in urban forests: Effects of habitat composition and connectivity
Source: Mov Ecol. 2016 Feb 17;4:5. doi: 10.1186/s40462-016-0071-z (PMC4758174; doi:10.1186/s40462-016-0071-z)

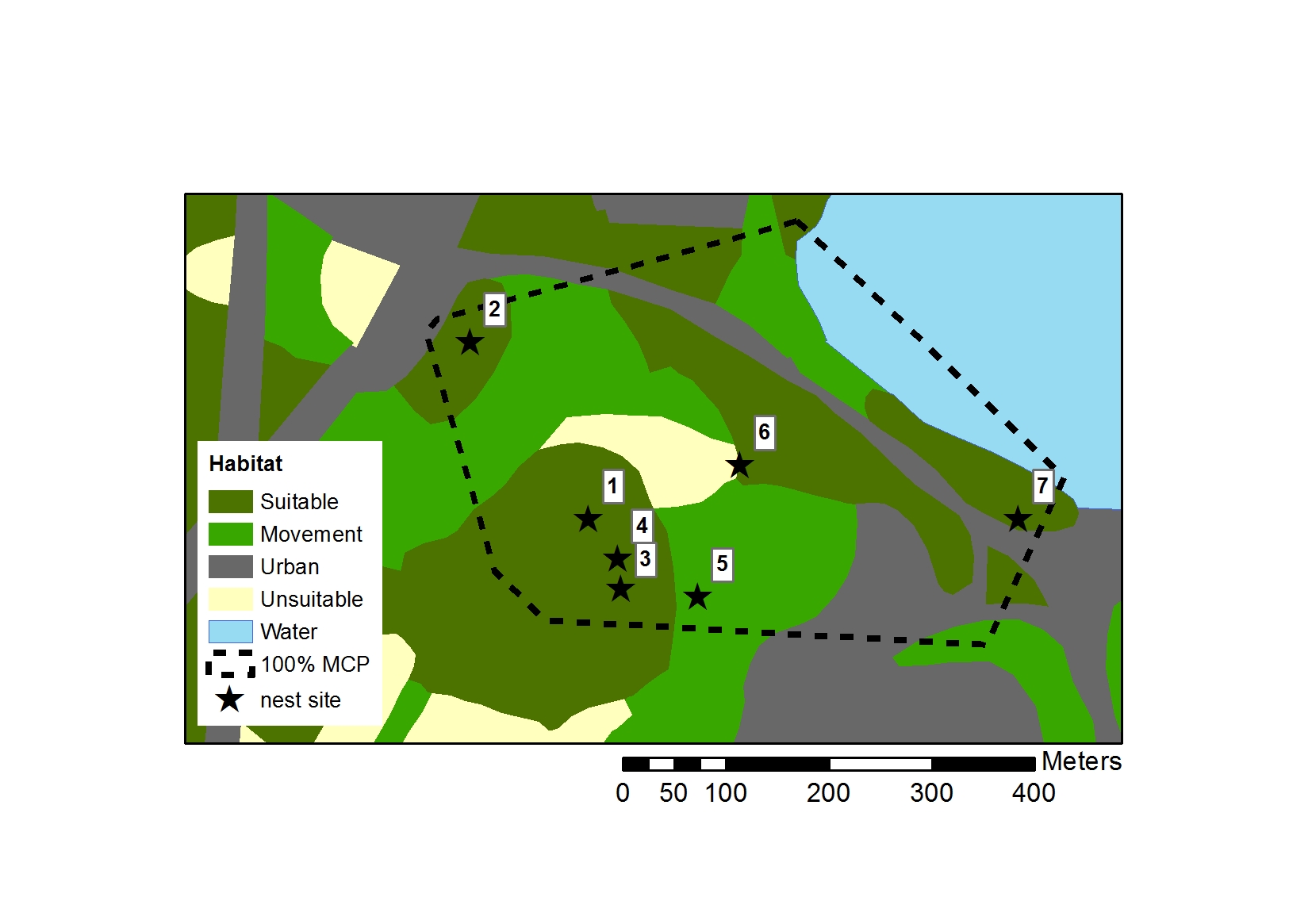

Supplement: Additional file 3: — Example of home-range habitat composition, and locations and connectivity of distinct nest sites of a female Siberian flying squirrel. A female home range by 100 % minimum convex polygon (MCP) is delineated by the black dashed line and numbered stars denote for the distinct nest sites. Different connectivity measures (see methods for details) are shown by blue arrowed lines. Individual could move from nest 1 to nest 2 by a straight line, or tortuously inside or outside home-range boundary, but in all cases the track would also comprise of movement habitat (connectivity measures C2, C4 and C6). However, if moving between nest sites 1, 3 and 4, all movements fall within the suitable habitat (connectivity measures C1, C3 and C5). In order to move from nest 6 to 7, female could move directly via suitable forest or taking detour, but it had to cross a gap in tree cover (connectivity measures C1g and C2g). (DOCX 862 kb) [file 40462_2016_71_MOESM3_ESM.docx]
