# Supplementary material for: Home-range use patterns and movements of the Siberian flying squirrel in urban forests: Effects of habitat composition and connectivity
Source: Mov Ecol. 2016 Feb 17;4:5. doi: 10.1186/s40462-016-0071-z (PMC4758174; doi:10.1186/s40462-016-0071-z)
Supplement: Additional file 4: — Results of model selection and comparison by Akaike’s Information Criteria (AIC). For each response variable (from A to D) a list of highest ranked models (within ΔAICc < 2) and their explanatory variables are shown. For every model the difference of AICc (AIC corrected for small sample sizes) from the best approximated model (lowest AIC) and Akaike weight (wi) is provided. (DOCX 13 kb) [file 40462_2016_71_MOESM4_ESM.docx]

| **Response variable** | **Model** | **ΔAIC*_c_*** | **w*_i_*** |
| --- | --- | --- | --- |
| A: length of burst | Sex + Month + H1 + H2 + H3 +Sex*Month | 0.00 | 0.29 |
|  | Sex + Month + H2 + H3 + Sex*Month | 0.17 | 0.27 |
|  | Sex + Month + H3 + Sex*Month | 0.60 | 0.22 |
|  | Sex + Month + H1 + H2 + H3 + Sex*Month + Sex*H2 | 1.83 | 0.12 |
|  | Sex + Month + H2 + H3 + Sex*Month + Sex*H2 | 1.97 | 0.11 |
| B: speed of burst | Sex + Month + H2 + H3 + Sex*Month | 0.00 | 0.19 |
|  | Sex + Month + H2 + H3 + Sex*Month + D(burst) | 0.28 | 0.16 |
|  | Sex + Month + H2 + H3 + Sex*Month + Sex*H3 | 0.91 | 0.12 |
|  | Sex + Month + H3 + Sex*Month | 1.00 | 0.11 |
|  | Sex + Month + H2 + H3 + Month*Sex + Sex*H3 + D(burst) | 1.38 | 0.09 |
|  | Sex + Month + H3 + Sex*Month + D(burst) | 1.42 | 0.09 |
|  | Sex + Month + H1 + H3 + Sex*Month | 1.62 | 0.08 |
|  | Sex + Month + H2 + H3 + Sex*Month + Sex*H2 | 1.83 | 0.07 |
|  | Sex + Month + H1 + H2 + H3 + Sex*Month | 1.90 | 0.07 |
| C: length of nightly track | Sex + Month + H2 + H3 + Sex*Month + Sex*H3 + D(night) | 0.00 | 0.31 |
|  | Sex + Month + H2 + Sex*Month + D(night) | 1.00 | 0.18 |
|  | Sex + Month + H1 + H2 + Sex*Month + Sex*H1 + D(night) | 1.50 | 0.14 |
|  | Sex + Month + H3 + Sex*Month + Sex*H3 + D(night) | 1.73 | 0.13 |
|  | Sex + Month + H1 + H3 + Sex*Month + Sex*H3 + D(night) | 1.83 | 0.12 |
|  | Sex + Month + Sex*Month + D(night) | 1.96 | 0.11 |
| D: number of nest sites | HR + E(sites) | 0.00 | 0.54 |
|  | HR + H2 + E(sites) | 1.67 | 0.24 |
|  | HR + H1 + E(sites) | 1.81 | 0.22 |
